# Acupuncture and Oxidative Stress

**DOI:** 10.1155/2015/424762

**Published:** 2015-03-19

**Authors:** Cun-Zhi Liu, Shu-Feng Zhou, Sergio-Botelho Guimarães, William Chi-shing Cho, Guang-Xia Shi

**Affiliations:** ^1^Acupuncture and Moxibustion Department, Beijing Hospital of Traditional Chinese Medicine Affiliated to Capital Medical University, 23 Meishuguanhou Street, Dongcheng District, Beijing 100010, China; ^2^Department of Pharmaceutical Sciences, College of Pharmacy, University of South Florida, 12901 Bruce B. Downs Boulevard, Tampa, FL 33612, USA; ^3^Department of Surgery, Federal University of Ceara (UFC), 60430-140 Fortaleza, CE, Brazil; ^4^Department of Clinical Oncology, Queen Elizabeth Hospital, Hong Kong

The current special issue is the 2014 issue which includes 7 interesting papers.

As one of the modalities of traditional oriental medicine, acupuncture has been widely used to treat many disorders and diseases including chronic pain, stroke, and insomnia as well as depression, while its mechanisms remain unclear. Enhanced production of reactive oxygen species causes oxidative stress leading to damage in lipids, proteins, and nucleic acids. Recent experimental studies have demonstrated that acupuncture could attenuate oxidative stress, which seems possible to explore the physiological antioxidative mechanism of acupuncture in various diseases.

This special issue contains 7 papers, of which 5 articles study the antioxidative mechanism of acupuncture in some diseases by animal models. These studies suggested that acupuncture may result from antioxidation, anti-inflammation, and antiapoptosis effects in several kinds of diseases. Among these, one study is related to the effect of laser acupuncture on memory impairment, oxidative stress status, and the functions of both cholinergic and dopaminergic systems in hippocampus of animal model of Parkinson's disease. One study explores whether electroacupuncture reduces myocardial ischemia-reperfusion (I/R) injury and inflammatory responses through inhibiting early growth response (Egr)-1 expression via the extracellular signal-regulated protein kinase-1 and kinase-2 (ERK1/2) pathway in a mouse model of myocardial ischemia reperfusion. Besides, one study compares the effects of antioxidant interventions on the electrical potential difference between acupoints along the stomach meridian on human. This paper suggests a possible underlying mechanism of acupuncture involving superoxide removal. One study focuses on the emerging links between acupuncture and redox modulation in vascular dementia, Alzheimer's vascular dementia, Parkinson's disease and hypertension, which represents an important step forward in the research of acupuncture antioxidative effect.

We are excited to explore the studies on the specific oxidative stress biomarkers and redox signaling cascades using oxidative stress-related assessments techniques should be particularly useful in generating new hypotheses to enhance our understanding of the mechanism of antioxidative effects of acupuncture.

*Cun-Zhi Liu* *Cun-Zhi Liu* *Shu-Feng Zhou* *Shu-Feng Zhou* *Sergio-Botelho Guimarães* *Sergio-Botelho Guimarães* *William Chi-shing Cho* *William Chi-shing Cho* *Guang-Xia Shi* *Guang-Xia Shi*

